# Rapid Determination of Chlorogenic Acid, Luteoloside and 3,5-O-dicaffeoylquinic Acid in Chrysanthemum Using Near-Infrared Spectroscopy

**DOI:** 10.3390/s19091981

**Published:** 2019-04-28

**Authors:** Zhengyan Xia, Yiming Sun, Chengyong Cai, Yong He, Pengcheng Nie

**Affiliations:** 1 College of Biosystems Engineering and Food Science, Zhejiang University, Hangzhou 310058, Zhejiang Province, China; zyxia@zju.edu.cn (Z.X.); alayy@zju.edu.cn (C.C.); 2 Zhejiang Research Institute of Traditional Chinese Medicine, Hangzhou 310023, Zhejiang Province, China; 15988855601@163.com; 3 State Key Laboratory of Modern Optical Instrumentation, Zhejiang University, Hangzhou 310027, Zhejiang Province, China

**Keywords:** Chrysanthemum, chlorogenic acid, luteoloside, 3,5-O-dicaffeoylquinic acid, near-infrared spectroscopy

## Abstract

The feasibility of near-infrared spectroscopy (NIR) to detect chlorogenic acid, luteoloside and 3,5-O-dicaffeoylquinic acid in Chrysanthemum was investigated. An NIR spectroradiometer was applied for data acquisition. The reference values of chlorogenic acid, luteoloside, and 3,5-O-dicaffeoylquinic acid of the samples were determined by high-performance liquid chromatography (HPLC) and were used for model calibration. The results of six preprocessing methods were compared. To reduce input variables and collinearity problems, three methods for variable selection were compared, including successive projections algorithm (SPA), genetic algorithm-partial least squares regression (GA-PLS), and competitive adaptive reweighted sampling (CARS). The selected variables were employed as the inputs of partial least square (PLS), back propagation-artificial neural networks (BP-ANN), and extreme learning machine (ELM) models. The best performance was achieved by BP-ANN models based on variables selected by CARS for all three chemical constituents. The values of r_p_^2^ (correlation coefficient of prediction) were 0.924, 0.927, 0.933, the values of RMSEP were 0.033, 0.018, 0.064 and the values of RPD were 3.667, 3.667, 2.891 for chlorogenic acid, luteoloside, and 3,5-O-dicaffeoylquinic acid, respectively. The results indicated that NIR spectroscopy combined with variables selection and multivariate calibration methods could be considered as a useful tool for rapid determination of chlorogenic acid, luteoloside, and 3,5-O-dicaffeoylquinic acid in Chrysanthemum.

## 1. Introduction

Chrysanthemum (the dry capitulum of *Chrysanthemum morifolium* Ramat) is a medicinal and edible cognate plant [1]. It was harvested in full bloom, and dried in the shade or over a fire, or dried in the sun after steaming. Modern studies show that Chrysanthemum has many biological and pharmacological characteristics including antibacterial, anti-inflammatory, antioxidant, vasodilator, hypolipidemic, and anti-tumor [2,3]. The Pharmacopoeia of the People’s Republic of China provides a standard of content of chlorogenic acid, luteoloside and 3,5-O-dicaffeoylquinic acid in Chrysanthemum [4]. Chlorogenic acid shows the activity of protecting the cardiovascular, antioxidation, antibacterial, antiviral, lipid-lowering, hypoglycemic, and liver protection [5,6]. Luteolin has anti-inflammatory, antiviral, and analgesic activity [7]. 3,5-O-dicaffeoylquinic acid is identified as an important chemical composition for antioxidant activity [8]. Chrysanthemum is widely distributed around the world.

The contents of chemical composition are different according to different environment, cultivars, harvest time, processing methods, and storage conditions [9,10]. The rapid determination of the contents of chlorogenic acid, luteoloside, and 3,5-O-dicaffeoylquinic acid are important for quality evaluation and clinical medication selection of Chrysanthemum.

Several techniques have been adopted for the determination of chlorogenic acid, luteoloside and 3,5-O-dicaffeoylquinicacid, including HPLC, gas chromatography–mass spectrometry and fluorescence spectrometry [11,12]. Nevertheless, these methods are expensive, time-consuming and require complicated sample preprocessing. Hence, it is necessary to develop a rapid and effective quantitative analysis method for the quality determination of Chrysanthemum.

With the advantages of being nondestructive, simple and fast, near-infrared spectroscopy (NIRS) has been widely applied in agriculture [13], the petroleum industry [14], food [15] and traditional Chinese medicine [16]. For instance, chlorogenic acid, caffeic acid, luteoloside, baicalin, ursodesoxycholic acid and chenodeoxycholic acid were analyzed in Tanreqing injection using FT-NIR, Tao applied NIR to determine the concentration of seven analytes including chlorogenic acid [17,18]. However, few studies are developed for quantitative analysis of chlorogenic acid, luteoloside and 3,5-O-dicaffeoylquinic acid in Chrysanthemum. 

In this work, a new method is proposed for the rapid determination of chlorogenic acid, luteoloside and 3,5-O-dicaffeoylquinic acid based on NIRS technology, which can provide components information for production, processing and the inspection of Chrysanthemum and its products.

## 2. Materials and Methods

### 2.1. Materials and Reagents

The reference substance of Chlorogenic acid, luteoloside, and 3,5-O-dicaffeoylquinic acid were derived from the National Institutes for the Foods and Drug Control (Beijing, China). The HPLC-grade acetonitrile was obtained from Tedia Scientific Inc. (Cincinnati, OH, USA). Methanol (analytical grade) and phosphoric acid (analytical grade, P85%) were purchased from Zhejiang Chemicals Company (Zhejiang, China). All other reagents were of analytical grade. Water used throughout the experiments was purified water provided by Wahaha Company (Zhejiang, China).

112 samples of Chrysanthemum were used in this research. Samples were provided by Zhejiang Research Institute of Traditional Chinese Medicine (Hangzhou, Zhejiang Province). Each sample was dried and grounded into powder, and only the powder which could pass through 0.25 mm pore mesh was used. These samples were kept in a temperature of 25 ± 2 °C and a moisture of 60 ± 10%. Among the prepared samples, 76 samples were selected randomly for calibration and the remaining 36 samples for independent prediction.

### 2.2. Spectrometric Measurements

Each sample was put in the sample cell and scanned by the Matrix Duplex NIR system working in the wavenumber range of 12,000 cm^−1^ to 4000 cm^−1^. All spectra were collected in a diffuse reflection mode with an optical fiber reflectance head and recorded as the log (1/R). Each sample was scanned 32 times and the average spectrum was regarded as the sample spectrum.

### 2.3. Reference Analysis Methods

The contents of chlorogenic acid, luteoloside, and 3,5-O-dicaffeoylquinic acid were determined by the HPLC method according to the Pharmacopoeia of the People’s Republic of China (2015 edition). The HPLC system was an Agilent 1100 series consisting of a vacuum degasser G1322A, a quaternary pump G1311A, an autosampler G1329A, a programmable variable wavelength detector (VWD) G1314B and a Thermostatted Column Compartment G1316A. The column was a Diamonsil C_18_ (250 × 4.6 mm, 5 µm) column. The detection wavelength was 348 nm. A gradient system was used consisting of two mobile phases. Mobile phase A was acetonitrile and the mobile phase B was 1% phosphoric acid solution. The gradient system was as follows: 0–11 min, 10–18% A, 11–30 min, 18–20% A, 30–40 min, 20% A. The flow rate was 1.0 mL/min, the injection volume was 5 µL.

### 2.4. Spectral Preprocessing

Before the calibration process, six preprocessing strategies were employed to reduce high-frequency random noise, baseline variation, path length differences and light scattering. The preprocessing methods include moving averages smoothing (MAS), Savitzky–Golay smoothing (SG), standard normal variate transformation (SNV), multiplicative scattering correction (MSC), the first derivative (1st-Der) and de-trending (Detrend). The results of different preprocessing methods were compared to choose the optimum preprocessing strategies. The preprocessing and calculations were carried out using the Unscrambler^®^ 10.1 software (Camo Process AS, Oslo, Norway).

### 2.5. Sensitive Variables Selection

A raw NIRS spectrum of a Chrysanthemum sample contained 2075 spectral bands, which suffered the collinearity and high dimensionality problems. In some cases, suitable methods can identify the most effective variables to reduce the input variables and improve the models’ accuracy and robustness [19,20]. Three methods for variables selection were used to reduce the collinearity and high dimensionality problems of NIR spectra and to develop simpler models. The methods include successive projections algorithm (SPA) [21], which could minimize variable collinearity, genetic algorithm-partial least squares regression (GAPLS) which combines the advantage of GA and PLS [22], and competitive adaptive reweighted sampling (CARS) [23,24,25]. CARS is a feature selection method combined Monte Carlo sampling with PLS regression coefficient.

### 2.6. Chemometric Calibration Method

Partial least square (PLS) algorithm [26] is a classic linear calibration method for spectral analysis. PLS extracts the main factors or sensitive variables (SV). Models are developed based on the scores of the main factors or SV according to their cumulative contribution rate [27,28]. 

Extreme learning machine (ELM) is one of learning neural algorithms, which has been successfully applied in nonlinear regression problems [29]. The algorithm randomly generates the connection weights between the input layer and the hidden layer. There is no need to adjust the threshold of the hidden layer neurons in the training process. The optimal solution is achieved when the number of hidden layer neurons is set. Comparing with traditional learning algorithms, ELM not only possesses the fast learning speed but also has a good generalization performance [30].

Backpropagation artificial neural network (BP-ANN) is one of the most popular neural network topologies [31]. BP-ANN extracts and establishes a complex correlation between inputs and outputs. The output represents the similarity between an object and a training pattern. As each process of the training pattern and the weight factor is adjusted, the difference between the calculated network output and the expected value is defined as the network output error, which will gradually decrease until the desired selection level is reached. An epoch is a one cycle through all training patterns [32,33].

### 2.7. Model Evaluation and Softwares

The performance of models was evaluated by five parameters including correlation coefficient of calibration (r_c_^2^), root mean square error of calibration (RMSEC), the correlation coefficient of prediction (r_p_^2^), root mean square error of prediction (RMSEP) and relative percent deviation (RPD). A good model should have higher r_c_^2^ and r_p_^2^ values, and lower RMSEC and RMSEP values. An RPD more than 1.5 is regarded as good predictions; an RPD between 2.0 and 2.5 indicates a satisfactory model for prediction, an RPD larger than 3.0 is considered as an efficient prediction model. In this study, r_p_^2^, RMSEP, and RPD were used as evaluation indexes to compare the models.

The spectral data extraction and the calculation of SPA, CARS, GA-PLS, ELM, and BP-ANN algorithms were performed by Matlab R2011a (The Math Works, Natick, MA, USA). PLS was conducted by Unscrambler^®^ 10.1 (CAMO AS, Oslo, Norway).

## 3. Results and Discussion

### 3.1. Features of NIR Spectra and HPLC Analysis

**Figure 1:**
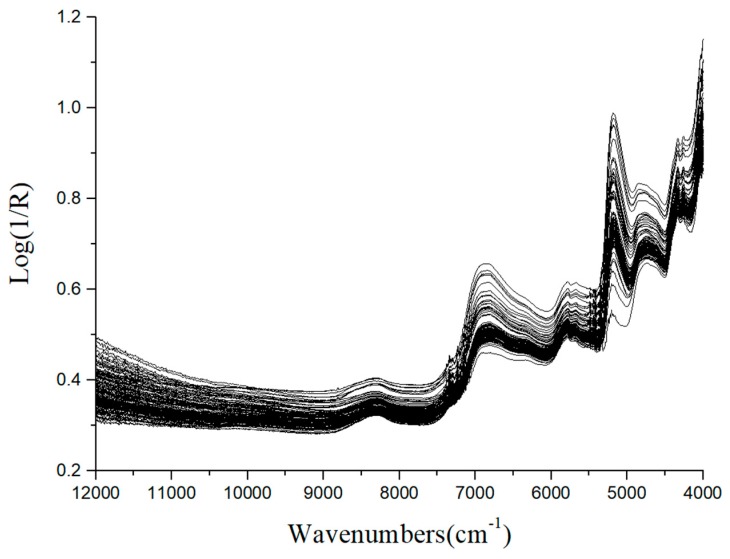
Raw spectra of Chrysanthemum measured by the Matrix Duplex NIR system working in the wavenumber range of 12,000 cm^−1^ to 4000 cm^−1^.

The original spectra of 112 Chrysanthemum samples are shown in Figure 1. It is noticed that the trends of all samples were quite similar except the different magnitudes of the spectra reflectance. This might be caused by different contents of chemical constituents of the samples, including chlorogenic acid, luteoloside and 3,5-O-dicaffeoylquinic acid. The reference values of chlorogenic acid, luteoloside and 3,5-O-dicaffeoylquinic acid in Chrysanthemum determined by HPLC are shown in Table 1. The content range of the measured components in the modeling set and the prediction set are similar, so, the established model can be representative.

### 3.2. Determination of the Best Preprocessing Algorithms

Different preprocessing methods were applied to the raw NIR spectra data. To identify the optimal preprocessing methods, PLS models were established based on different pretreated spectra data as input variables. Results of the PLS models based on the raw and pretreated spectra data are shown in Table 2. The best result was obtained based on SG pretreated spectra for the chlorogenic acid prediction. The prediction results of the SG model had a good r_p_^2^ value of 0.843 and a small RMSEP value of 0.047. The optimal PLS model (r_p_^2^ = 0.741 and RMSEP = 0.033) was achieved by the SNV preprocessing for the luteoloside prediction. For the 3,5-O-dicaffeoylquinic acid prediction, the best performance with r_p_^2^ of 0.843 and RMSEP of 0.072 was obtained by the raw spectra data. The best input variables were employed for further calculation.

### 3.3. Sensitive Variables Selection

**Figure 2:**
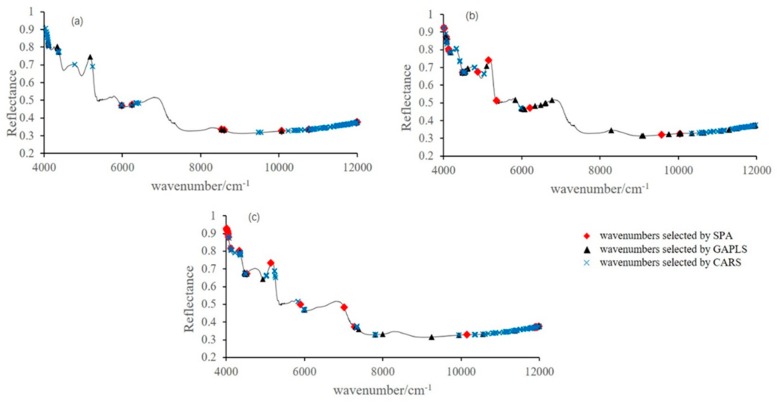
Sensitive variables selected by different methods for prediction of (**a**) chlorogenic acid, (**b**) luteoloside, and (**c**) 3,5-O-dicaffeoylquinic acid.

The data of raw NIRS spectrum of Chrysanthemum contained 2075 bands. These bands had collinearity and high dimensionality problems. To reduce the input variables and the collinearity problems, SPA, GA-PLS, and CARS were compared. Shown from Figure 2, by applying these methods, the number of variables decreased from 2075 to less than 106. The selected variables simplified the model and improved the speed of the computation process.

### 3.4. Model Calibration

Three kinds of regression models (PLS, ELM, BP-ANN) were established based on the selected SVs as input variables. The performance of each model is shown in Table 3. Compared with full spectral PLS models (Table 2), the CARS-PLS model had a better performance with r_p_^2^ of 0.899, RMSEP of 0.038 and RPD of 3.184 for the prediction of chlorogenic acid. The SPA-PLS and GAPLS-PLS models had similar results, compared with the full spectral model. The results indicated that CARS was an effective variable selection method and SVs identified by CARS contained the most relevant and representative information. SPA and GAPLS also obtained positive results, as only 0.3% and 1.4% of the full spectral bands were selected as input variables, and their models also had a good prediction. Moreover, BP-ANN models achieved better performance than PLS and ELM models and the best prediction performance was obtained by CARS-BP-ANN model (processed by SG), which had r_p_^2^ of 0.924, RMSEP of 0.033 and RPD of 3.667. 

For the luteoloside content prediction, the GAPLS-PLS and CARS-PLS models showed better results than the full spectral PLS model, indicating that the SVs selected by GAPLS and CARS extracted the most useful information to represent the full spectra. Compared with the full spectra model, similar performance was achieved based on the variables selected by SPA, which had only 0.6% of full variables. Therefore, SPA was also regarded as a helpful way of variable selection. Similar to the chlorogenic acid analysis, BP-ANN models were better than the corresponding PLS and ELM models. The best result was obtained by the BP-ANN model based on the variables selected by CARS, and the best model had r_p_^2^ of 0.927, RMSEP of 0.018 and RPD of 3.667.

**Figure 3:**
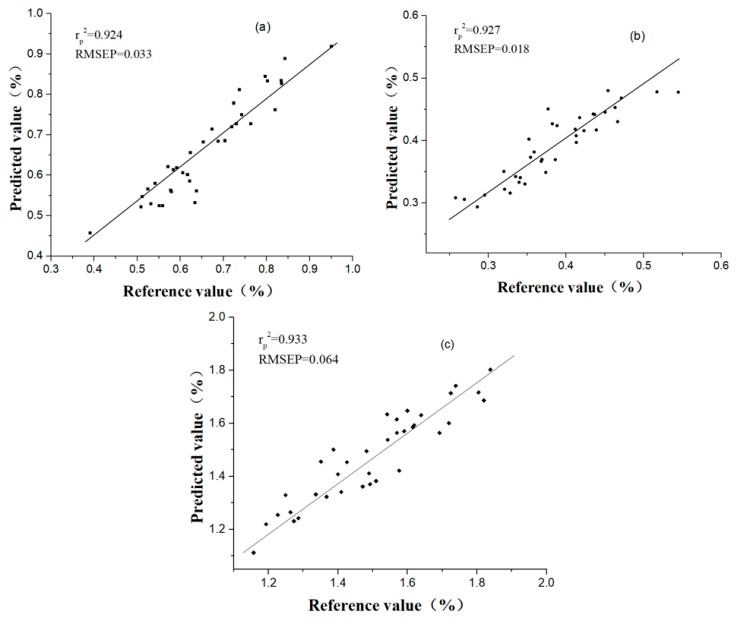
Results of CARS-BP-ANN models in the prediction set for prediction of (**a**) chlorogenic acid, (**b**) luteoloside, and (**c**) 3,5-O-dicaffeoylquinic acid.

For 3,5-O-dicaffeoylquinic acid prediction, compared with the full spectral PLS models, GAPLS-PLS and CARS-PLS models obtained better performance, which implied that GAPLS and CARS were effective variable selection methods. Although the value of r_p_^2^ of the SPA-PLS model was lower than the PLS model based on full spectra, SPA was still considered as a useful method, since the number of input variables of SPA-PLS model decreased 99.0%, from 2075 to 20, whereas the r_p_^2^ value only reduced 8.5%. Same to the prediction of chlorogenic acid and luteoloside, BP-ANN models also achieved the best performance. The best prediction results were obtained by the CARS-BP-ANN model, which had r_p_^2^ of 0.933, RMSEP of 0.064, and RPD of 2.891. Therefore, BP-ANN was considered as the optimal calibration method for the prediction of chlorogenic acid, luteoloside and 3,5-O-dicaffeoylquinic acid in Chrysanthemum. The scatter plots of the best results of CARS-BP-ANN models in the prediction set for prediction of chlorogenic acid, luteoloside and 3,5-O-dicaffeoylquinic acid are shown in Figure 3.

## 4. Conclusions

In this work, the feasibility of NIR spectroscopy for the rapid determination of chlorogenic acid, luteoloside and 3,5-O-dicaffeoylquinic acid contents in Chrysanthemum was explored. Different preprocessing, variable selection and regression methods were employed and their results were compared. SG and SNV were considered as the optimal preprocessing method for the prediction of chlorogenic acid and luteoloside respectively and raw data was the best for the prediction of 3,5-O-dicaffeoylquinic acid. SPA, GAPLS and CARS were proposed to recognize sensitive variables which were important to predict chlorogenic acid, luteoloside and 3,5-O-dicaffeoylquinic acid contents. The BP-ANN models achieved better performance than PLS and ELM models and the best performance was achieved by the BP-ANN models based on variables selected by CARS for the prediction of all three chemical constituents in Chrysanthemum. The best spectral models for the prediction of chlorogenic acid, luteoloside and 3,5-O-dicaffeoylquinic acid had r_p_^2^ of 0.924, 0.927, 0.933, RMSEP of 0.033, 0.018, 0.064, RPD of 3.667, 3.667, 2.891, respectively. The above results indicated that NIR spectroscopy combined with variables selection and multivariate calibration methods has the potential to be considered as a useful tool for the rapid determination of chlorogenic acid, luteoloside and 3,5-O-dicaffeoylquinic acid in Chrysanthemum. In the future, more Chrysanthemum samples with a wider range of chlorogenic acid, luteoloside and 3,5-O-dicaffeoylquinic acid contents should be considered to further improve the accuracy, robustness and adaptability of models for industrial application.

## Figures and Tables

**Table 1:** Referent contents of Chlorogenic acid, luteoloside, and 3,5-O-dicaffeoylquinic acid in Chrysanthemum determined by HPLC.

	Calibration Set			Prediction Set		
	Range (%)	Mean (%)	S.D. ^1^	Range (%)	Mean (%)	S.D.
Chlorogenic acid	0.388–0.961	0.648	0.121	0.390–0.950	0.660	0.121
Luteoloside	0.255–0.552	0.388	0.063	0.258–0.545	0.387	0.066
3,5-O-dicaffeoylquinic acid	0.985–1.839	1.506	0.189	1.193–1.838	1.499	0.185

^1^ Standard deviation.

**Table 2:** Results of PLS models with different data preprocessing methods.

Quality	Preprocessing	Number of Latent Variables	Calibration		Prediction				
			r_c_^2^	RMSEC	r_p_^2^	RMSEP	Slope	Bias	RPD
Chlorogenic Acid	None	9	0.906	0.036	0.797	0.054	0.775	−0.014	2.241
	MAS	6	0.841	0.047	0.841	0.047	0.727	−0.011	2.574
	SG	6	0.839	0.047	0.843	0.047	0.728	−0.010	2.574
	SNV	7	0.876	0.041	0.762	0.059	0.756	−0.017	2.051
	MSC	9	0.878	0.041	0.767	0.057	0.768	−0.017	2.123
	1-Der	5	0.882	0.040	0.740	0.061	0.738	−0.009	1.984
	Detrend	7	0.869	0.042	0.808	0.052	0.773	−0.014	2.327
Luteoloside	None	13	0.976	0.009	0.728	0.034	0.753	0.004	1.941
	MAS	11	0.910	0.018	0.738	0.033	0.734	−0.004	2.000
	SG	11	0.901	0.019	0.741	0.033	0.754	0.001	2.000
	SNV	12	0.974	0.010	0.741	0.033	0.761	0.005	2.000
	MSC	10	0.949	0.014	0.728	0.034	0.748	0.004	1.941
	1-Der	8	0.918	0.018	0.650	0.039	0.624	−0.006	1.692
	Detrend	11	0.964	0.012	0.691	0.036	0.731	0.003	1.833
3,5-O-dicaffeoylquinic acid	None	10	0.920	0.053	0.843	0.072	0.842	0.012	2.569
	MAS	10	0.918	0.054	0.832	0.075	0.838	0.014	2.467
	SG	10	0.876	0.066	0.815	0.078	0.793	0.011	2.372
	SNV	8	0.846	0.073	0.766	0.088	0.754	−0.004	2.102
	MSC	9	0.908	0.057	0.823	0.077	0.831	−0.002	2.403
	1-Der	5	0.803	0.083	0.605	0.114	0.791	−0.003	1.623
	Detrend	9	0.918	0.054	0.814	0.079	0.843	0.015	2.342

**Table 3:** Results of different regression methods based on sensitive variables for chlorogenic acid, luteoloside, and 3,5-O-dicaffeoylquinic acid.

Quality	Preprocessing	Variable Selection Methods	Models	Calibration		Prediction		
				r_c_^2^	RMSEC	r_p_^2^	RMSEP	RPD
Chlorogenic Acid	SG	SPA	PLS	0.859	0.044	0.843	0.047	2.574
			ELM	0.846	0.046	0.876	0.047	2.574
			BP-ANN	0.889	0.039	0.857	0.047	2.574
		GAPLS	PLS	0.910	0.035	0.841	0.048	2.521
			ELM	0.878	0.041	0.834	0.052	2.327
			BP-ANN	0.885	0.039	0.874	0.044	2.750
		CARS	PLS	0.970	0.020	0.899	0.038	3.184
			ELM	0.972	0.020	0.882	0.041	2.951
			BP-ANN	0.964	0.022	0.924	0.033	3.667
Luteoloside	SNV	SPA	PLS	0.803	0.027	0.736	0.033	2.000
			ELM	0.856	0.023	0.738	0.033	2.000
			BP-ANN	0.834	0.025	0.783	0.031	2.129
		GAPLS	PLS	0.801	0.027	0.759	0.032	2.063
			ELM	0.648	0.036	0.733	0.036	1.833
			BP-ANN	0.846	0.024	0.814	0.028	2.357
		CARS	PLS	0.976	0.009	0.910	0.020	3.300
			ELM	0.998	0.000	0.819	0.041	1.610
			BP-ANN	0.955	0.013	0.927	0.018	3.667
3,5-O-dicaffeoylquinic acid	None	SPA	PLS	0.808	0.082	0.771	0.087	2.126
			ELM	0.843	0.074	0.808	0.083	2.229
			BP-ANN	0.848	0.074	0.850	0.072	2.569
		GAPLS	PLS	0.869	0.068	0.856	0.069	2.681
			ELM	0.904	0.058	0.878	0.065	2.846
			BP-ANN	0.906	0.058	0.908	0.056	3.304
		CARS	PLS	0.974	0.031	0.927	0.049	3.776
			ELM	0.949	0.042	0.893	0.064	2.891
			BP-ANN	0.962	0.039	0.933	0.064	2.891
